# Accelerated bone growth, but impaired implant fixation in allograft bone mixed with nano-hydroxyapatite - an experimental study in 12 canines

**DOI:** 10.1186/s40634-022-00465-z

**Published:** 2022-04-23

**Authors:** Lau Lind Petersen, Jørgen Baas, Mette Sørensen, Joan E. Bechtold, Kjeld Søballe, Jeppe Barckman

**Affiliations:** 1 Department of Orthopedics, Aarhus University Hospital Skejby, Palle Juul-Jensens Boulevard, 99 8200 Aarhus N, Denmark; 2 Departments of Orthopaedic Surgery and Biomedical Engineering, University of Minnesota Life Sciences, Building 700 South 10th Avenue, Minneapolis, MN 55415 USA

## Introduction

Overcoming bone loss is a common challenge during revision of failed prostheses, and impaction-grafting is a well-established method to counter this problem [14, 28]. The gold standard of bone graft is autograft bone [12], but it has several disadvantages including donor site morbidity and limited amounts for grafting larger defects [34]. Although allograft bone is inferior to autograft bone in terms of osteoinduction and osteogenicity [13], allograft is most often chosen due to its higher availability, known osteoconductivity and absence of donor site morbidity [24, 32].

The demand for primary hip prostheses rising markedly as the population ages [11]. With a 10% risk of revision within the first decade of primary hip replacement [17], the absolute number of people who require revision of failed hip prostheses also thereby is expected to increase. Furthermore, revision prostheses have higher failure rates, with up to 26% risk of failure within the first 10 years [27]. Failures are mostly due to aseptic loosening, which is thought to be related to reduced osseointegration and early implant subsidence due to bone loss that prevents adequate initial fixation [10, 29].

Nano-hydroxyapatite (nHA) is widely used in dentistry and orthopedics as a bone substitute or bone graft extender [20, 30]. Several studies have shown nHA to have osteoconductive and some osteoinductive properties [2, 8, 9, 15, 18–21, 25, 26]. Tibia plateau defects have been filled with nHA with promising results regarding bone healing [20]. An in vitro experiment has demonstrated that the physical presence of nHA does not impede the mechanical stability of the impacted graft material at concentrations of 10% nHA. At 33% nHA, however, the mechanical stability seems to decline [2].

To our knowledge, the combination of morselized allograft bone and nHA have never been studied regarding osseointegration of grafted implants in vivo. Ostim® which is a paste of 35% volume nHA particles and 65% water was used for this study.

The aim of the study is to evaluate the effect of nHA augmented graft on implant fixation. It was hypothesized, that the nHA would serve as a conductor for new bone ingrowth within the grafted defect, thereby increasing bone growth and thus improving implant fixation.

## Methods and materials

### Design, location and ethical approval

**Fig. 1:**
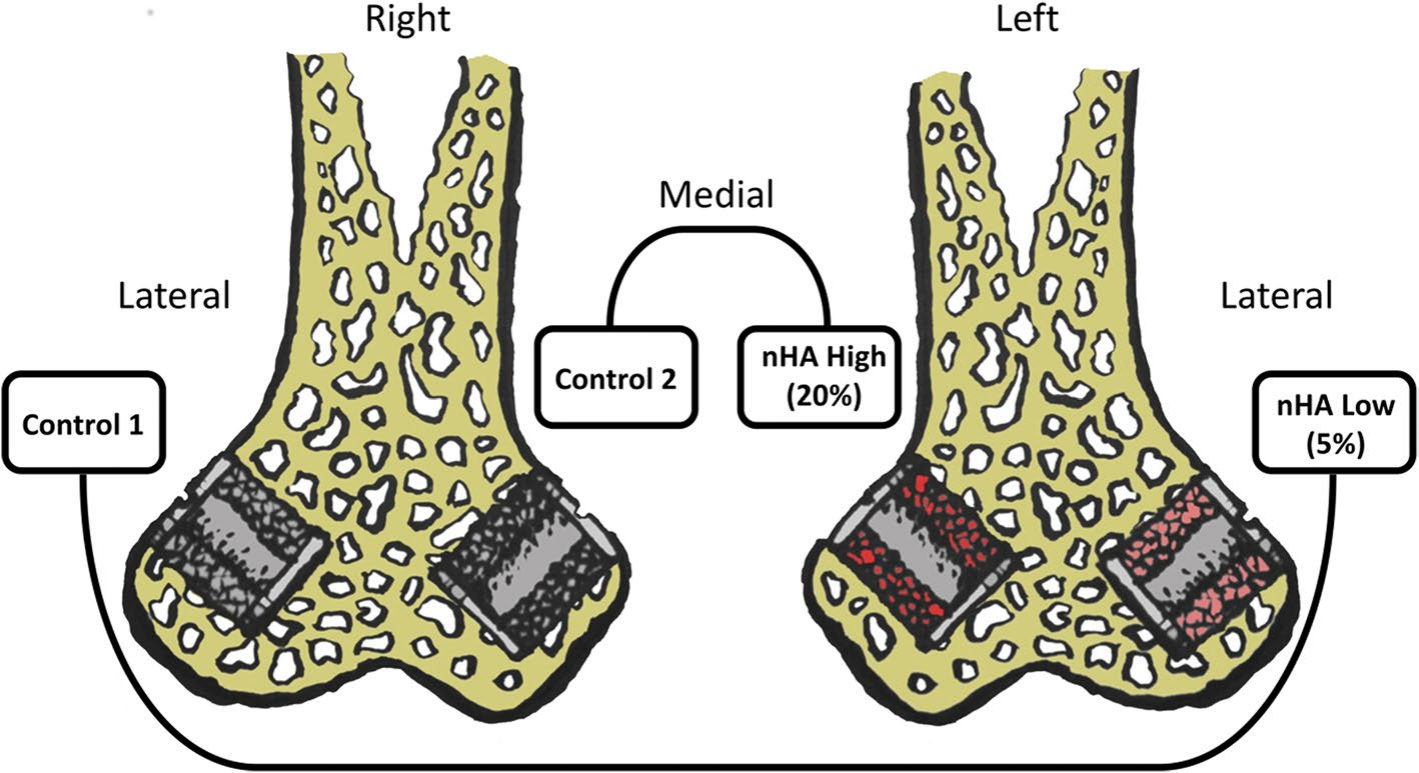
Implants in distal femur

This experiment was conducted as a double-paired animal study. A well-established peri-implant gap model [31] was employed in which impacted allograft bone with or without addition of nHA was compared. A total of 12 skeletally mature Class A American Hounds were included with a mean age of 11 (10–15) months and a mean weight of 32 (29–36.) kg. The hounds were bred for scientific purposes and had individual cages. They were fed ad-libitum standard dog food (21% Lab Dog Diet #8755, Teklad Diets, Madison WI). Two additional animals were used as allograft bone donors. For this study, each animal received four implants, two in each distal femur (Fig. 1). Surgery and post-operative care were carried out at a facility in Minneapolis, licensed with USDA and AAALAC. This study was approved by the International Animal Care and Use Committee.

**Fig. 2:**
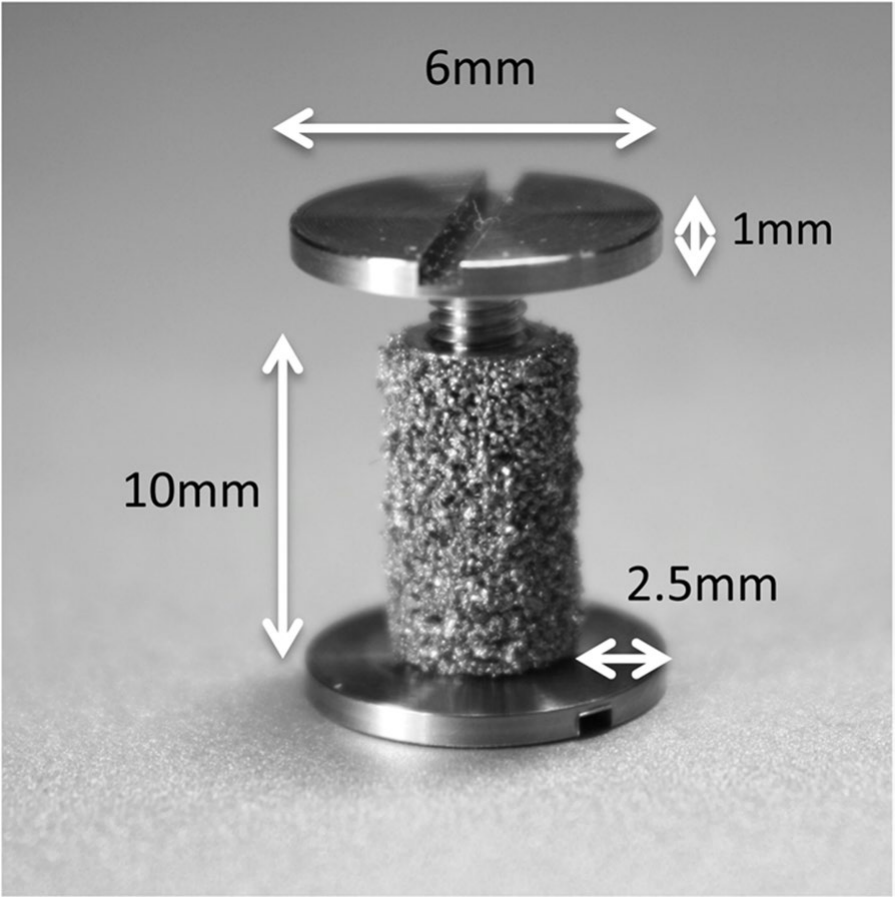
Titanium implant

A total of 48 titanium alloy implants (Ti-6A1-4 V) were used with a porous coating used on commercially available hip prostheses (manufactured and donated by DePuy Inc. (Warsaw, IN, USA)). The implants consist of a solid cylindrical core with a height of 10 mm and a diameter of 6 mm. An 11-mm end-screw was attached in both ends (Fig. 2). This created a 2.5 mm concentric peri-implant gap around the core of the implant when inserted into an 11-mm drill hole. The gap around the control implants were impacted with morselized allograft bone. In the intervention groups the bone graft impacted into the gap was augmented with either a low dose (5% of volume) nHA (nHA Low) or high dose (20% of volume) nHA (nHA High). There were two paired groups. In the lateral condyle: nHA Low vs. Control 1 and in the medial condyle: nHA High vs. Control 2 (Fig. 1). This way, only contralateral condyles were compared in order to rule out any potential difference between the lateral and the medial condyle. The groups were also randomly distributed between left and right.

### Allograft bone

Bone graft was harvested under sterile conditions from two donor animals not included in this study. The proximal humeri, the proximal tibiae and the distal femora were used. The donor animals were not genetically connected to the recipients, thus ensuring non-histocompatibility as in the clinical situation with human allograft. Prior to preparation, all soft tissue and cartilage was removed, and the bones were morselized using a standard bone mill (Biomet**®**, Warsaw, IN, USA) creating bone chips of 1–3 mm in size.

The volume of bone in the 2.5 mm circumferential gap of this model is 0.67 cm^3^. Results from prior studies show that approximately 1.38 g of morselized bone can be impacted into this gap [4]. Sixteen portions of bone graft were prepared for each of the four experimental groups. A total of 89 g bone graft (64 portions of 1.38 g) was rinsed by gentle stirring in 890 ml saline at a temperature of 37 °C for 1 min and repeated three times with fresh saline. The rinsed bone graft was divided into four groups, each group sub-divided into 16 equal portions (4 of which were extras in case needed) and stored in sterile double-containers at − 80 °C. The bone graft was rinsed to remove excess fat and loose connective tissue, enabling an addition of a larger volume of nHA-paste to the bone graft.

### Preparation of the nHA bone graft mixture

Ostim® was used (Heraeus Kulzer Nordic AB, Kolding, Denmark) as a pH neutral source of nHA. Ostim® is a mouldable paste and provides no structural stability itself [18]. Having the same inorganic components as human bone, nHA has been shown to have high osteoconductivity. The size of the nHA particles (100x20x3 nm) are shown to accelerate neovascularization [25].

To facilitate mixing of the nHA in bone graft, the Ostim® paste was diluted by adding sterile saline in a 1:1 ratio. To ensure an even dispersion of the nHA into the allograft bone, the substances were mixed twice. Firstly, the allograft bone from each group was divided into 16 samples, to which nHA was added. Secondly, all 16 samples from each group were pooled, and mixed thoroughly. Finally, the groups were divided into 16 portions again, stored in double sterile containers, and frozen at − 80 °C.

### Surgery

Under general anaesthesia and sterile conditions, the femoral epicondyles were exposed through medial and lateral incisions. The periosteum was divided and loosened from the bone with a rongeur. A 2.5-mm guide wire was inserted perpendicular to the surface of the epicondyle 18 mm from the distal edge of the condyle and 14 mm from the anterior edge of the condyle. A drill speed of maximum two rotations per second was used to avoid thermal tissue damage. The drill hole was 11 mm wide and 12 mm deep. The implant with its 2.5 mm end-screw attached was inserted into the drill hole. With a specially designed hollow impaction tool, the peri-implant gap was impacted with bone graft. To ensure homogeneous distribution of the graft in the defect, one third of the graft was impacted into the bottom third of the gap, then one third of the graft was impacted into the middle third of the gap, and finally, the remaining graft was impacted into the most superficial third of the gap. Impaction was done by hand force and by the same surgeon. Finally, the top screw was screwed into the threaded implant core, and the incision closed in layers. Following closure, Bupivacaine 0.5% was administered locally at the incision site. Postoperatively, each animal received a transdermal Fentanyl patch (75 μg/h) for three days. The animals rested in separate cages and were allowed unrestricted weight bearing and daily exercise. Observation time was four weeks. Euthanasia was performed with acepromazine (0.5 mg/kg), an anaesthetic dose of propofol (4 mg/kg), followed by an injection of hypersaturated barbiturate. Afterwards, the distal femora were harvested, cleaned and frozen at − 80 °C. One surgeon performed all surgeries.

### Specimen preparation

**Fig. 3:**
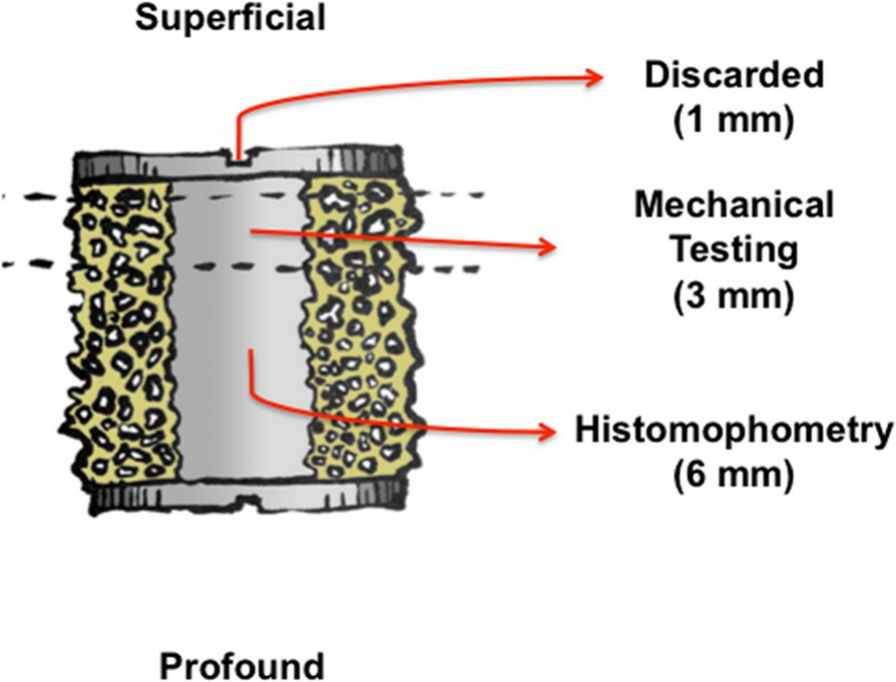
Sections of the implants for mechanical and histomorphometric testing

Implant-bone specimen blocks were cut out of the bone on a water-cooled diamond band saw (Exact Apparatebau, Nordenstedt, Germany). The top-screw and the most superficial 1 mm of the implant were removed and discarded. The remaining 9 mm of the implant was divided into two sections. The outermost 3 mm was stored at − 20 °C pending the mechanical push-out test. The remaining 6 mm (Fig. 3) was dehydrated in graded alcohol (70%, 96% and 100%), 2-propanol, and xylene and embedded in methylmethacrylate (Art. 800,590, Merck, Darmstadt, Germany), and prepared for the histomorphometric analysis.

For the histomorphometry, each specimen was rotated around its vertical axis prior to sectioning. This produced randomly defined sectioning planes. Using vertical sectioning technique [3] four 30-μm sections were cut on a glycerol-cooled microtome (KDG-95- MeProTech, Heerhugowaard, The Netherlands). Each section was surface stained with 0.1% toluidine blue (pH = 7, Fluka, Sigma-Aldrich, St. Louis, MO, USA) for 10 min, rinsed and mounted on glass.

### Mechanical testing

**Fig. 4:**
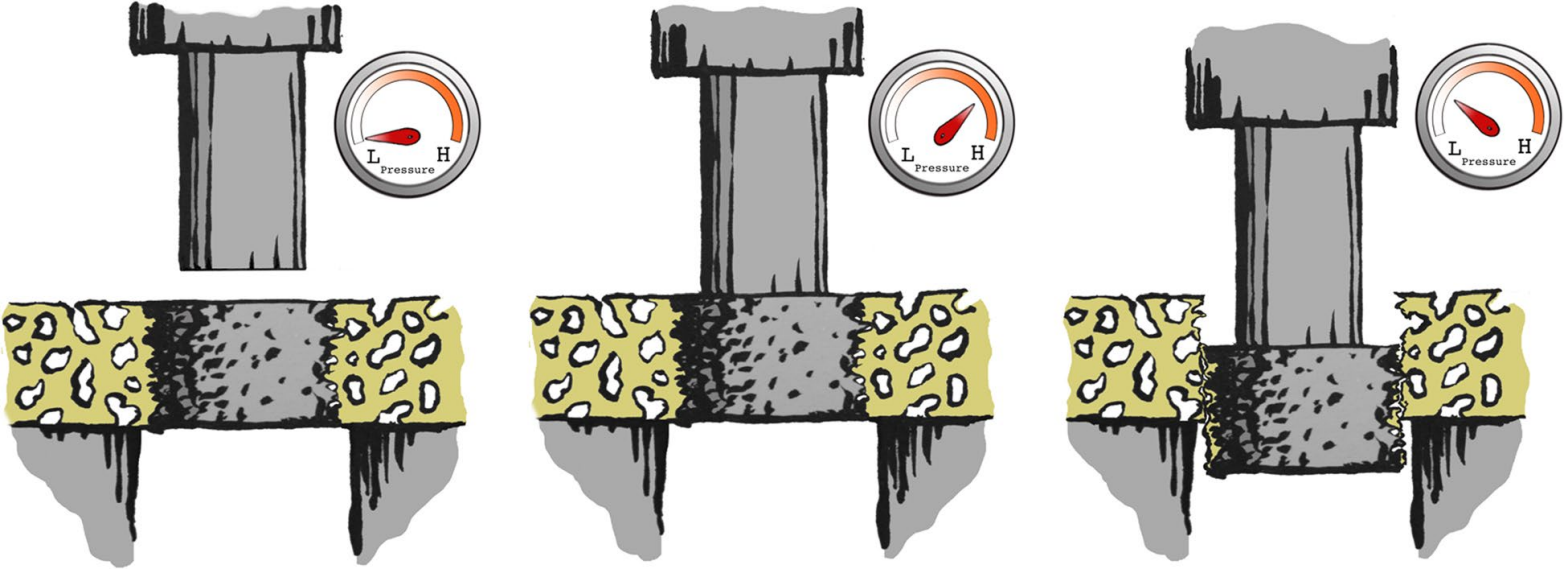
Mechanical push-out test. The specimen is placed on a metal platform with a central opening. Pressure is measured to evaluate the strength and stiffness of the osseointegration of the implant

We conducted the mechanical testing on an MTS Bionics Test Machine (MTS 858 Mini Bionix, MN, USA, Software: MTS Test Star 790.00 Version 4.0C). The test was performed blinded and in one session. Each specimen was placed over a 7.4-mm hole and a 5-mm cylindrical test probe was used. With a preload of 2 N and at a rate of 5 mm/min, the implant was pushed out of the surrounding tissue (Fig. 4). Load versus displacement data was continuously recorded and the mechanical parameters (maximum shear strength, apparent shear stiffness, and total energy absorption) were derive d[31].

### Histomorphometric analysis

**Fig. 5:**
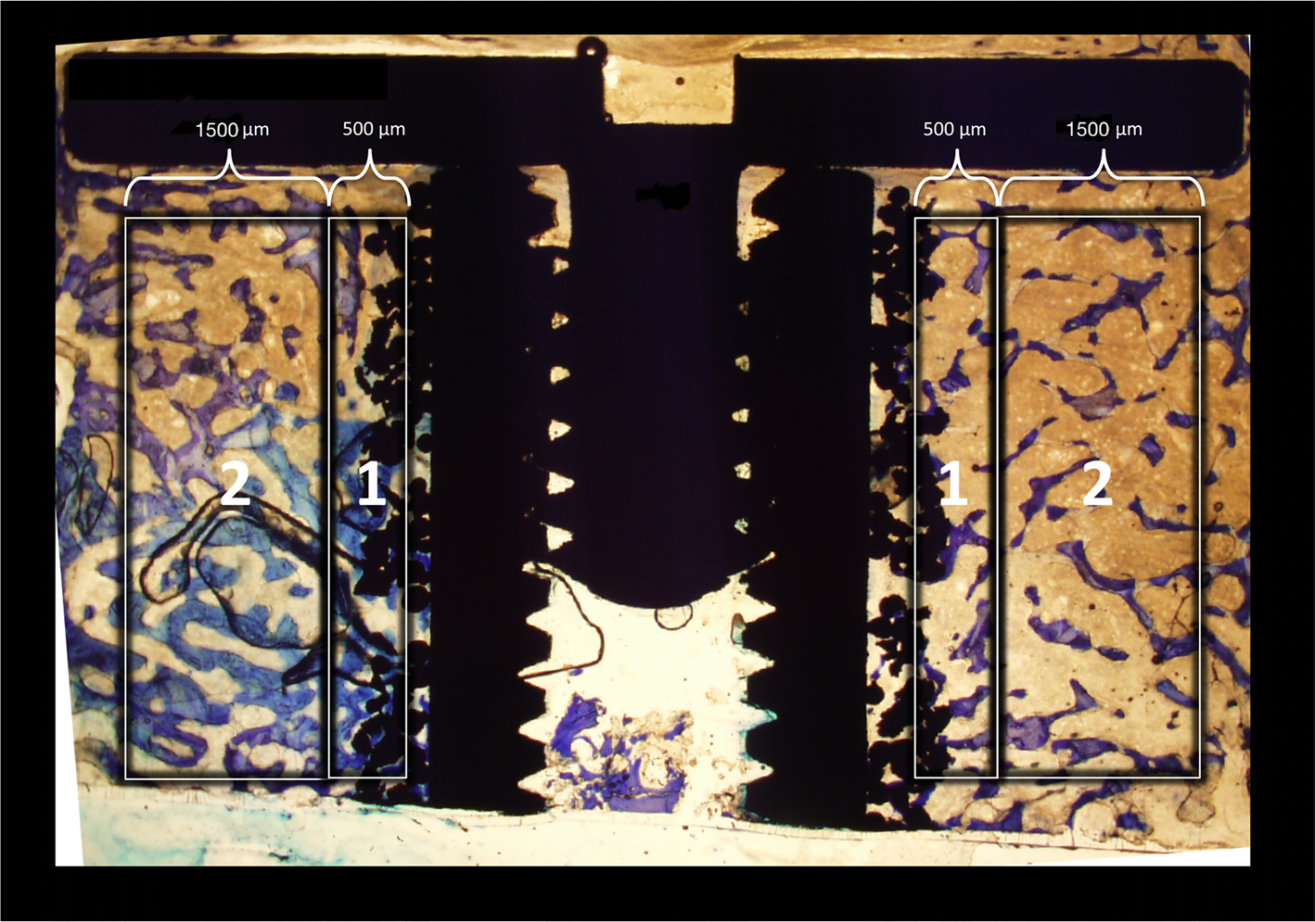
Shows the two regions of interest for the histomorphometric analysis. The Inner Gap (1) covers the first 500–1000 μm. The Outer Gap (2) is 1500 μm wide

**Fig. 6:**
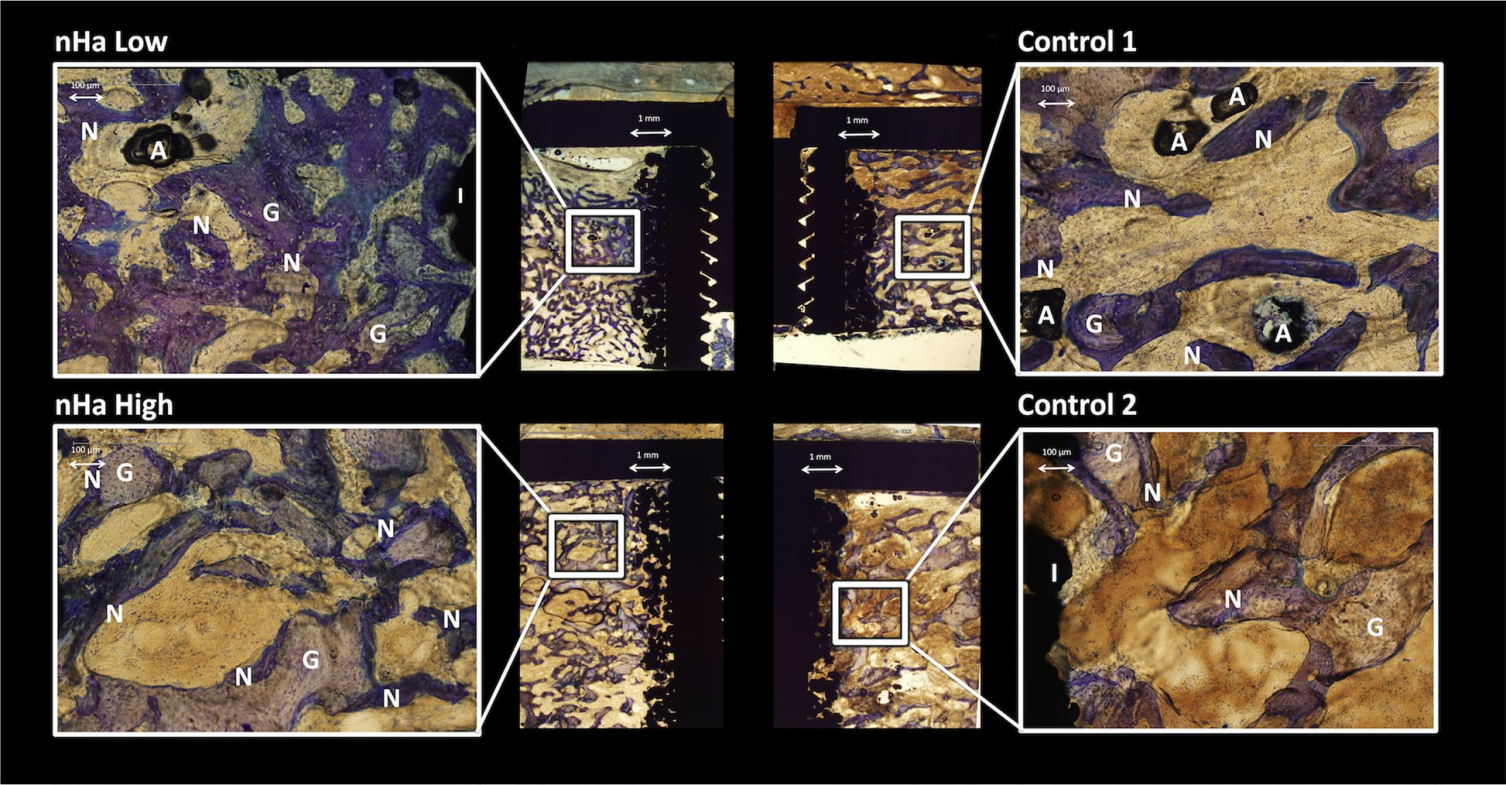
Representative photos of histomorphometry showing interventions vs controls. Implant (I), new bone (N), graft bone (G), artefacts (A)

On each histological section, blinded histomorphometric analysis was performed using 10x magnification. The stereological software was used C.A.S.T Grid (newCAST-version 3.4.1.0; Visiopharm A/S, Horsholm, Denmark). Two regions of interest were defined: The Inner Gap ranging from the mid part of the porous coating of the implant to 500 μm into the gap and the Outer Gap, ranging from 500 to 2000 μm further into the gap (Fig. 5). Volume fractions were quantified by point-counting technique [16] and surface ongrowth was quantified by line intercept technique [3]. New bone, fibrous tissue, graft bone, and bone marrow space were assessed (Fig. 6). Osteoid was included as new bone.

### Statistics

Stata 11.2 (StataCorp, College Station, TX, USA) was used for statistical analyses. Mechanical data were normally distributed as evaluated by q-norm plots of the residuals. The histomorphometric data were normally distributed, except for volume and surface ongrowth of fibrous tissue. All parametric data was analyzed with a two-tailed Student’s t-test. Results are presented as mean with standard deviation (SD). For non-parametric data, we used the Wilcoxon Signed Rank test and the results are presented as median with interquartile range (IQR). Statistical significance was considered for *p*-values less than 0.05.

## Results

All animals recovered fully, but two weeks postoperatively one animal needed debridement of the knee joint due to superficial infection. The consulting veterinarian performed an open lavage of the knee joint but left the implants untouched and it healed uneventfully. Another animal needed re-suture of the superficial layers. Otherwise, all animals healed uneventfully.

**Fig. 7:**
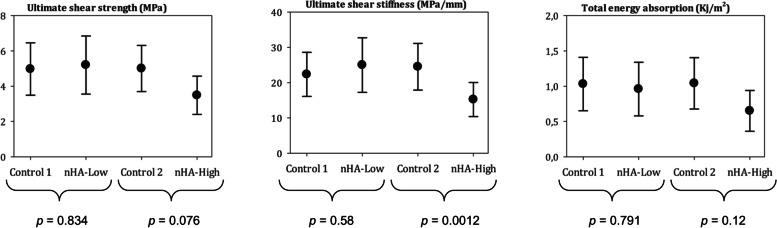
Ultimate Shear strength, ultimate shear stiffness and total energy absorption measured during the push-out test. [mean (95% CI)]

For all mechanical parameters, the Control implants were comparable to the Low dose nHA implants. The High-dose nHA implants had statistically significantly inferior stiffness compared to the Control implants (mean 15.2 vs 24.5) *p* = 0.01; Fig. 7). Histomorphometrically, a relative 45% increase in new bone volume and a relative 44% increase in new bone surface coverage was found when comparing the nHA High group to control (Table 1). No statistically significant differences were found when the nHA low group was compared with its control regarding osseointegration (Table 2).

**Table 1:** Histomorphometric results of Control 1 vs nHA Low, SD standard deviation, IQR interquartile range

	New bone mean % (SD)	Fibrous tissue median % (IQR)	Bone graft mean % (SD)
*Implant surface*			
Control 1	22 (9.9)	2 (0–9)	1 (0.4*2*)
nHA Low	27 (12.*7*)	4 (0–14)	1 (0.3*8*)
t-test	*p = 0.17*		*p = 0.27*
Wilcoxon		*p = 0.53*	
*Inner gap (0–500* μm*)*			
Control 1	29 (12)	2 (0–6)	7 (2.9)
nHA Low	34 (0.13)	1 (0–9)	6 (3.5)
t-test	*p = 0.25*		*p = 0.42*
Wilcoxon		*p = 0.45*	
*Outer gap (500–2000 μm)*			
Control 1	25 (6.4)	0 (0–1)	14 (3.9)
nHA Low	30 (6.2)	0 (0–1)	14 (4.4)
t-test	*p* ***=*** *0.06*		*p = 0.89*
Wilcoxon		*p = 0.91*

**Table 2:** Histomorphometric results of Control 2 vs nHA High, SD standard deviation, IQR interquartile range

	New bone mean % (SD)	Fibrous tissue median % (IQR)	Bone graft mean % (SD)
*Implant surface*			
Control 2	18 (6.5)	1 (0–8)	1 (1.1)
nHA High	26 (7.9)	3 (0–5)	1 (0.6)
t-test	***p = 0.011***		*p = 0.56*
Wilcoxon		*p = 0.97*	
*Inner gap (0–500 μm)*			
Control 2	25 (7.3)	1 (0–5)	7 (3.6)
nHA High	31 (7.1)	0 (0–6)	8 (4.1)
t-test	***p = 0.048***		*p = 0.48*
Wilcoxon		*p = 0.72*	
*Outer gap (500–2000 μm)*			
Control 2	20 (5.2)	0 (0–1)	14 (4.7)
nHA High	29 (6.2)	0 (0–1)	16 (4.6)
t-test	***p = 0.001***		*p = 0.197*
Wilcoxon		*p = 0.48*	

## Discussion

In this study, we found that augmenting morselized allograft bone with nHA paste increased new bone formation, but weakened the fixation of titanium implants at a high dose of nHA.

The implant model is a well-established model of a large peri-implant gap. It has been used for many years and enabled the evaluation of both the mechanical fixation and osseointegration of the same implant. The simplicity of this implant model secures a high degree of variance control. However, it has some limitations. The grafted implants were not influenced by direct load or joint fluid. Additionally, surgery was performed on young healthy animals with a bone quality not compromised by the presence of a failed primary implant. Dogs were chosen due to their close resemblance in bone biology to human bone [1].

The hypothesis of this study was adding nHA to the bone graft would increase its osteoconductivity and osteoinductivity leading to improved new bone formation and thereby improved implant fixation. Ostim® was chosen as the source of nHA due to its mouldable consistency and high biocompatibility [18], but other formulations may not behave the same. The small nHA particles may have been able to partially fill the empty space between the allograft bone pieces without increasing the total volume of the impacted bone graft, hence the graft amount was not changed among groups. Laterality was assumed to possibly influence healing, so only medial right and left implants were compared with each other, as well as lateral right and left implants were compared. The observation period of 4 weeks was chosen based on previous experience with graft resorption and new bone formation in the same animal model [6].

Augmenting bone graft with nHA led to increased bone formation. This biologic response was observed both in the grafted region surrounding the implant as well as directly on the implant surface. The formation of new bone was statistically significantly higher for the High-dose nHA group, but not for the Low-dose group.

The purpose of stimulating new bone formation is to achieve a solid mechanical anchorage of the implant in living bone to sustain implant stability, durability and survival. However, no improvement of the mechanical fixation of the implants was found in the nHA-augmented implants. In the High-dose nHA group, a statistically significant decrease in mechanical implant fixation was observed.

The phenomenon of increased bone formation and weakened mechanical implant fixation has been observed in many previous experiments seeking to stimulate new bone formation [6]. Many of these experiments have shown that increased new bone formation is coupled to an increased resorption of bone graft [7] as well as resorption of readily formed new bone leading to a transient weakening of the implant anchorage pending remodelling to lamellar bone [22]..

In the current experiment with nHA no changes in the amounts of bone graft between the groups were found, rendering the above explained phenomenon unlikely in this case. A longer observation period may have resulted in a more complete remodelling towards lamellar bone yielding a more rigid fixation. The observation period of 4 weeks was chosen to evaluate the effect on early implant fixation as this correlates to later implant loosening [23].

Arts et al. [2] mixed nHA with bone grafts in a study with two separate models, one testing mechanical strength in vitro, another measuring histological bone growth in vivo on rabbits. At 10% nHA, mechanical strength increased, and at 33% increased bone growth was observed in the rabbit model. Mechanical strength, however, decreased at 33% nHA. Their study design did not allow both mechanical and histomorphometric analysis on the same specimen. Also, no titanium implants were used. With the current study design, it was possible to analyze bone growth and mechanic stability around a porous-coated titanium implant. A 5% addition of nHA was chosen for the nHA Low group and 20% for the High group. Accordingly, a four-fold difference between the high dose and the low dose intervention was seen with the high dose below their 33% nHA. These results show an increased bone growth, but at the cost of mechanical strength with 20% nHA (again with the caveat that there was only one observation period, at 4 weeks). This concurs with Arts and coworkers’ while maintaining site-specific control [2].

A reason for the impaired mechanical fixation observed in the high dose group may have been related to the 20% dose of nHA. The study was designed as a doubled paired study in order to reduce biological differences between medial and lateral femur epicondyle. For that reason, a middle group of 10% volume concentration could not be included, while maintaining site-specific control. It is unknown if 10% volume concentration would have favoured osseointegration without hampering the mechanical stability.

Although several studies have reported promising effects on bone healing with the use of nHA particles [19, 21], some have found remnant particles of nHA embedded in bone or fibrous tissue, even after longer periods of observation (up to 8 months) [15, 5]. Others have shown cell toxicity caused by nHA particles in lung and kidney tissue [33]. Another concern with the use of nHA augmented bone graft is the possibility that the nHA particles may cause wear on arthroplasty prostheses. These factors were not evaluated in the current model, but the concerns should be considered for eventual clinical use.

## Conclusion

Within the limits of this model, this study found that adding nHA to allograft bone around a cylindrical implant results in increased new bone formation around the implant but with decreased mechanical anchorage of the implant in the surrounding bone. Early mechanical implant fixation is important to achieve good long-term implant survival. For this reason, it is premature to recommend this method in implant grafting procedures of joint replacements until further studies have been conducted evaluating different concentrations. It may, however, be of interest in other gap-filling grafting procedures in which early mechanical stability is not a surgical goal, since longer observation periods may improve the mechanical properties of the woven bone.

## Abbreviation

nHA: Nano-hydroxyapatite
